# Swallow Magnetic Resonance Imaging Compared to 3D-Computed Tomography for Pouch Assessment and Hiatal Hernias After Roux-en-Y Gastric Bypass

**DOI:** 10.1007/s11695-020-04758-z

**Published:** 2020-06-21

**Authors:** Daniel M. Felsenreich, Michael A. Arnoldner, Felix B Langer, Christoph Bichler, Natalie Vock, Katharina Steinlechner, Mahir Gachabayov, Aram Rojas, Dietrich Beitzke, Thomas Mang, Gerhard Prager, Christiane Kulinna-Cosentini

**Affiliations:** 1 Department of Surgery, Division of General Surgery, Vienna Medical University, Waehringer Guertel 18-20, 1090 Vienna, Austria; 2 Department of Biomedical Imaging and Image-guided Therapy, Vienna Medical University, Vienna, Austria

**Keywords:** Roux-en-Y gastric bypass, 3D-volumetry, Swallow MRI, Weight regain

## Abstract

**Introduction/Purpose:**

Weight regain and weight loss failure after bariatric surgery are important issues that may require a weight regain procedure. Three-dimensional-computed tomography (3D-CT) is a well-established method allowing exact measurements of pouch volume. The aims of this study were to prove the applicability of swallow MRI as a non-ionizing procedure and compare it to 3D-CT in patients after weight regain procedures following RYGB.

**Materials and Methods:**

Twelve post-RYGB patients who had a follow-up operation for weight regain before 12/2017 were included in this prospective study. Swallow MRI and 3D-CT were performed in each patient to evaluate the size of the anastomosis, pouch volume, and intrathoracic pouch migration (ITM).

**Results:**

Mean pouch volume in swallow MRI and 3D-CT were 40.4 ± 21.0 ml and 43.5 ± 30.2 ml, respectively (*p* = 0.83), and pouch diameter at the maximal distention was 35.3 ± 5.9 ml (MRI) and 31.0 ± 10.0 ml (CT) (*p* = 0.16). The rate of ITM was 75% in both examinations (*p* = 1.0).

**Conclusion:**

Swallow MRI is a valid method for the assessment of pouch volume in different phases of the swallowing process and is comparable to 3D-CT. The diagnosis of ITM using swallow MRI was equal to 3D-CT.

## Background

The number of bariatric surgical procedures performed worldwide increases every year and exceeded 685,000 in 2016. Roux-en-Y Gastric Bypass (RYGB) was the most common procedure until 2014, when sleeve gastrectomy (SG) was more commonly performed. More than 7% of all bariatric procedures are revisional operations due to weight regain [1]. There is a consensus that obesity is to be considered a chronic disease and some patients may need more than one bariatric procedure to achieve sufficient long-term weight loss [2].

As revisional surgery is (and will increasingly be) an important issue, elucidating the reason for weight loss failure, and weight regain is a major concern [3]. Following RYGB, both of these issues are often associated with a dilated pouch, a remnant fundus at the top of the pouch after incomplete mobilization of the fundus, or a widened anastomosis [4]. Thus, the most commonly used examination tools are gastroscopy [4, 5], oral contrast swallow, and [6] 3D-swallow computed tomography volumetry (3D-CT) [7, 8].

Swallow MRI, a new imaging modality for the assessment of the postoperative gastroesophageal junction, has gained increasing interest in the literature [9, 10]. Swallow MRI can provide additional information about the size of the pouch in a functional manner during the process of swallowing. Nevertheless, swallow MRI in bariatric patients has not been described in the literature as yet.

The aims of this study were to prove the applicability of swallow MRI in bariatric patients as a valid non-ionizing alternative to 3D-CT and even highlight its advantages. Both examinations were thus compared in patients after weight regain procedures following RYGB, with respect to pouch volume and detection of intrathoracic pouch migration (ITM).

## Materials and Methods

Twelve RYGB patients who had a follow-up operation for weight regain before 12/2017 were included in this prospective study. The procedures performed for weight regain were pouch resizing, pouch banding, or combinations of both. Pouch resizing was performed only if a dilatation of the pouch was detected in the preoperative gastroscopy. The band was placed loosely in all patients during pouch banding, 2 cm above the gastrojejunostomy. Patients were asked for GERD symptoms at the time of RYGB, at the time of the weight regain procedure, and before the 3D-CT and swallow MRI examinations.

All patients included in this study underwent 3D-CT and swallow MRI after the weight regain procedure. None of the examinations had to be interrupted or discontinued. Patients who were unable to swallow large volumes of liquids at a time, as well as patients with claustrophobia and pregnant patients, had been excluded. Both examinations were performed at the same institute specifically for the current study, with a maximum time between studies of 2 weeks.

The ethics committee of the Vienna Medical University approved this study (EK 2262/2017). All patients were covered by insurance and written informed consent was obtained from each patient.

### 3D-Computed Tomography

Three-dimensional-CT in the current study was performed on a 384-row scanner (Somatom Force, Siemens Healthineers, Erlangen, Germany). Each patient was examined with the same protocol described below.

Patients were instructed to fast for 4 h prior to the CT scan and were then administered 10 mg butylscopolaminiumbromide orally 2 h before the scan. Patients were asked to position themselves on the scanner table in a supine left anterior oblique position and a topogram was obtained. The scan region was established and 500 ml of an oral water-soluble iodinated contrast solution (Iopamidol, Gastromiro®, Bracco, Vienna, Austria; in a dilution 1:10 with tab water) was administered. Patients were told to drink the solution as quickly as possible, and, just after finishing, they received one pack of effervescent sodium bicarbonate. Subjects were instructed to avoid burping to optimize the distension of the gastric pouch. The scan was performed immediately after in a craniocaudal direction during inspiration without additional IV contrast media administration. The following scan parameters were used: x-ray tube voltage, 120 kV; automatic tube current modulation, gantry rotation speed, 0.5 s; and beam collimation 192 × 0.6 mm. A PACS workstation (IMPAX software) was used for CT interpretation and postprocessing was completed with SyngoVia (Siemens Healthineers, Erlangen, Germany).

For volumetry, the pouch was identified, with the gastrojejunostomy considered its distal margin. The gastroesophageal junction was considered the proximal end of the staple line, as the angle of His was dissected free in all patients. The length of the pouch was measured from the gastrojejunostomy to the proximal end of the staple line, regardless of whether it ended above or below the diaphragm. The pouch was examined in multiple planes and its margins were outlined. The complete volume of the pouch was evaluated by adding together the parts of the pouch filled with air and those filled with contrast.

Intrathoracic pouch migration was diagnosed if staple lines were visible above the hiatus. Finally, the diameter of the gastrojejunostomy was measured, as well as the maximum short axis pouch diameter on a parasagittal oblique reconstruction.

### Dynamic Swallowing MRI

MR imaging was performed on a 1.5-T MRI scanner (Ingenia, Philips Medical Systems, Best, Netherlands) with a phased array coil placed upon the patient’s chest. Prior to the actual exam, the clinical history was obtained by the radiologist and the patient’s ability to swallow in the supine position was tested outside of the MRI scanner. The MRI protocol used in this study is shown in Table 1.

**Table 1:** MRI protocol

Sequence	Voxel size (mm)	Slice thickness (mm)	Flip angle	TR (ms)	TE (ms)
T2 TSE axial	1.4 × 1.6 × 3.0	3	90°	448	80
dyn. bFFE sagittal	1.7 × 1.9 × 5.0	5	60°	3.26	1.63
dyn. bFFE axial	1.7 × 1.9 × 5.0	5	60°	3.55	1.78
dyn. bFFE coronal	1.7 × 1.9 × 5.0	5	60°	3.49	1.75

*TR* repetition time, *TE* echo time, *bFFE* balanced fast field echo sequence, *TSE* turbo spin echo sequence, *ms* milliseconds, *mm* millimeter

Patients were placed on the scanner table in the supine left anterior oblique (LAO) position and an axial T2-weightened turbo spin echo sequence (TSE) was obtained to depict the gastric pouch and gastroesophageal junction. To determine the optimum slice angle of the dynamic evaluation, a sagittal oblique balanced fast field echo sequence (bFFE) was centered at the long axis of the pouch in accordance with the T2w TSE. The bFFE sequence was then performed dynamically in the paracoronal, parasagittal, and paraxial planes, with contiguous slices beyond the outline of the pouch for full coverage and to compensate for plane displacement due to respiratory or swallowing motions and pouch distension. For pouch distention, a cup filled with water was placed close to the patient’s head in the MR gantry. The patients were instructed to drink the whole cup (250 ml) via a long plastic tube while scanning. This was performed in each of the aforementioned planes.

MRI interpretation was conducted on a PACS Workstation (IMPAX, Agfa-Gevaert) by M.A., who was blinded to the results of 3D-CT if performed prior to the MRI. The maximum short-axis pouch diameter before and at full distension was noted in the parasagittal plane. Volumetric analysis was performed with commercially available postprocessing software (QMass, Medis; Leiden, Netherlands), with the dynamic “cine” bFFE sequence.

### Statistical Analysis

IBM SPSS Statistics for Windows (v 25.0) was used for all statistical computations. Metric data in the current study are described using mean ± SD, if approximately normally distributed or median [min, max], if highly skewed. Categorical data are represented as absolute frequency/sample size (percentages). For the assessment of rater agreement, a two-way mixed model intraclass correlation coefficient (ICC) was used for absolute agreement. To compare 3D-CT and MRI results, Students’ *t* tests or Mann-Whitney *U* tests were applied. A *p* value ≤ 0.05 was considered to indicate significant results. No multiplicity corrections were performed in order to prevent an increasing error of the second type.

## Results

The present study includes a total of 12 patients (100% female), all of whom had a RYGB and an operation because of weight regain at the Vienna Medical University. One patient had an SG before and was converted to a RYGB. Procedures for weight regain after RYGB were pouch resizing in two patients (16.7%), pouch banding in nine patients (75%), and a combination of both in one patient (8.3%). Weight and BMI at the time of RYGB was 140.2 ± 20.9 kg and 50.4 ± 7.0 kg/m^2^ and 108.2 ± 19.4 kg and 38.9 ± 6.7 kg/m^2^ at the time of the weight regain procedure, respectively. The interval between the RYGB and the weight regain procedure was 69.0 ± 29.1 months, on average. Within the end of the follow-up (57.8 ± 38.2 months after RYGB), the patients were able to decrease their weight and BMI to 93.0 ± 17.1 and 33.4 ± 6.0 (Table 2).

**Table 2:** Patient characteristics (*n* = 12)

	All patients (*n* = 12)
Sex (female) (%)	100
Before RYGB	
Median age OP (years)	36.7 ± 11.1
Weight (kg)	140.2 ± 20.9
BMI (kg/m^2^)	50.4 ± 7.0
Interval RYGB–WR procedure (months)	69.0 ± 29.1
Before WR procedure	
Weight (kg)	108.2 ± 19.4
BMI (kg/m^2^)	38.9 ± 6.7
After WR procedure	
Mean follow-up (months)^a^	57.8 ± 38.2
Weight (kg)	93.0 ± 17.1
BMI (kg/m^2^)	33.4 ± 6.0

*OP* operation, *BMI* body mass index, *EWL* excess weight loss, *RYGB* Roux-en-Y gastric bypass, *WR* weight regain

^a^Referring to the WR procedure

None of these patients was suffering from reflux after the RYGB, but three patients (25%) had reflux postoperatively after the pouch banding and are now on proton pump inhibitors to treat their symptoms. Two (16.7%) had temporary dysphagia, but the band did not have to be removed in any of them.

### 3D-CT Volumetry

The 3D-CT volumetry results from all 12 patients included a mean pouch volume of 43.5 ± 30.2 ml. Further, the mean short-axis pouch diameter at its maximal distention during swallowing was 31.0 ± 10.0 mm and the mean diameter of the anastomosis was 13.0 ± 4.0 mm. Seventy-five percent (nine patients) had staples above the diaphragm, which was interpreted as intrathoracic pouch migration. In terms of radiation exposure, the mean dose-length product in the 3D-CT scans was 282.6 ± 149.7 mGy**/**cm (Table 3).

**Table 3:** 3D volumetry CT and swallow MRI (*n* = 12)

	3D-CT	MRI	*p* value
Pouch volume pre-swallow (ml)	–	9.5 ± 5.5	–
Pouch volume post-swallow (ml)	43.5 ± 30.2	40.4 ± 21.0	0.83
Diameter anastomosis (mm)	13.0 ± 4.0	13.1 ± 3.6	0.76
Max. pouch diameter pre-swallow (mm)	–	15.9 ± 5.2	–
Max. pouch diameter post-swallow (mm)	31.0 ± 10.0	35.3 ± 5.9	0.16
DLP (mGy**/**cm)	282.6 ± 149.7	–	–
Pouch migration	75%	75%	1.00

*RYGB* Roux-en-Y gastric bypass, *DLP* dose-length product, *3D-CT* three-dimensional computed tomography, *MRI* magnetic resonance imaging

### Swallow MRI

**Fig. 1:**
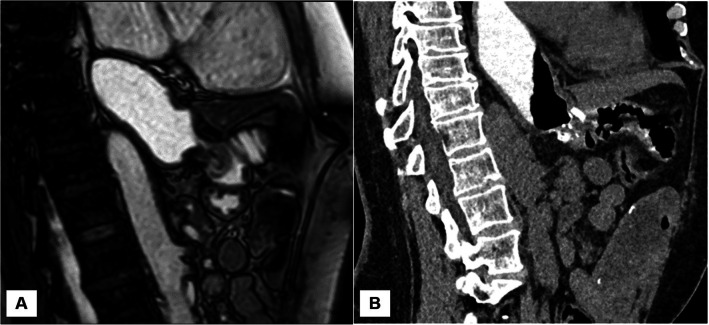
A 30-year-old female who underwent RYGB, pouch resizing, and banding; parasagittal dynamic bFFE MRI (**a**) and reconstructed 3D-CT (**b**) demonstrate the distended pouch. There is mild ITM

**Fig. 2:**
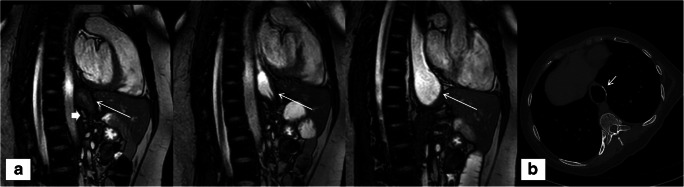
A 50-year-old female who underwent RYGB, pouch resizing, and banding (**a** short, bold arrow); dynamic MRI shows filling of the pouch during fluid intake (**a** long arrows). Moderate ITM was suspected and confirmed by CT (**b** short arrow indicates staple lines above the diaphragm)

Results of the swallowing MRI as a dynamic examination in the same 12 patients showed a mean pouch volume before the liquid bolus of 9.5 ± 5.5 ml and of 40.4 ± 21.0 ml at maximum pouch expansion (Figs. 1 and 2). The mean diameter of the anastomosis was 13.1 ± 3.6 mm. Before liquid bolus ingestion, the mean short-axis diameter of the pouch was 15.9 ± 5.2 mm, and after the liquid bolus, it proved to be 35.3 ± 5.9 mm. In the swallowing MRI, the same nine patients (75%) were again identified with intrathoracic pouch migration (Table 3).

Comparing the two examinations of 3D-CT and swallow MRI in terms of pouch volume (*p* = 0.83), the diameter of the pouch in its maximal distension (*p* = 0.16), the diameter of the anastomosis (*p* = 0.76), and pouch migration (*p* = 1.00), the results were quite comparable and no statistically significant differences were identified (Table 3).

## Discussion

The current study presents comparable results of 3D-CT and swallow MRI in terms of pouch volume, diameter of the pouch/anastomosis, and rates of ITM. The pouch volume measured in swallow MRI before administering any liquid was more than four times smaller than at maximal pouch distention in this study. The exact volume measured at maximal distention in swallow MRI (with water) was quite similar to the volume measured in 3D-CT (with oral contrast and effervescent granules) after swallowing. Thus, this study demonstrates that swallow MRI has no disadvantage in the reproducibility of measurements compared to 3D-CT.

To the authors’ best knowledge, there is no study available in the literature on swallow MRI in RYGB patients as yet. Only one prior study reported pouch measurements with MRI in patients who received adjustable gastric banding (AGB) [11]. However, this was performed statically without pouch distension. In contrast, the presented series of patients confirms the feasibility of swallow MRI as a dynamic examination in patients after RYGB.

While there are no studies on swallow MRI in RYGB patients available at this point, a small number of studies have reported the use of swallow MRI in patients with normal gastric situs [10], after laparoscopic sleeve gastrectomy (LSG) [12], after fundoplication [9, 13], or to evaluate specific diseases of the esophagus/pharynx [14].

To evaluate the role of dynamic swallow MRI for the assessment of gastroesophageal reflux and esophageal motility disorders in patients with normal gastric situs, a study with 37 GERD (gastro-esophageal reflux disease) patients found that the results of 24-h pH-metry and manometry were concordant in 82% of patients with reflux and 67% of patients with esophageal motility disorders. Further, the authors reported swallow MRI to be particularly suitable for the evaluation of the persistence and the size of hiatal hernias [10]. Also, Hosseini A. et al. studied the detection of hiatal hernias with swallow MRI and endoscopy in 107 patients with GERD symptoms. In 79% of patients, hiatal hernias were detected at either one or both examinations, while 25% of hiatal hernias were detected only during a Valsalva maneuver in swallow MRI. These results lead to the conclusion that both examinations were complementary in the detection of hiatal hernias [15].

### Advantages and Limitations of Swallow MRI

The aforementioned studies highlight the fact that swallow MRI is a feasible examination in patients with normal situs and diseases of the pharynx and esophagus, as well as for the evaluation of the postoperative gastroesophageal junction. The advantages of using this examination in a bariatric surgical setting have, however, not been addressed in the literature, as yet. The current study shows the feasibility of this examination with results quite comparable to those of 3D-CT.

Does swallow MRI add any value to pouch evaluation in bariatric patients compared to 3D-CT? First, it should be noted that swallow MRI does not include any radiation. Further, swallow MRI facilitates observation of the whole swallowing process, as it is a dynamic examination. Thus, the examiner is able to focus on the diameter of the pouch when it is entirely filled with liquid in order to measure the real maximal diameter of the pouch. In particular, the evaluation of very short pouches may benefit from this fact, as liquid may pass quickly in a short pouch, and can, therefore, present with inadequate distension on 3D-CT. Finally, the dynamic nature of swallow MRI may prove very useful in patients with sliding hernias suffering from dysphagia, as the examination will reveal the manner in which the pouch is emptied.

Limitations of swallow MRI for pouch evaluation are that the accuracy of the measured volumes is lower than those in 3D-CT. Due to the dynamic process and the longer acquisition time, MR volumetry is less precise than 3D-CT. ITM is easily detected on CT when staple lines are located above the diaphragm. Although staples are generally better visible on CT than on MRI, a hiatal hernia detection rate of up to 85% is described in the literature for MRI [9] and all hernias were correctly identified on MRI in the present study. Another disadvantage of swallow MRI compared to static 3D-CT is that the swallowing process must take place in the supine position, which may not be possible in every patient.

In terms of costs, MRI is usually more expensive than CT. The reimbursement by (public) health insurance varies considerably for each examination internationally. In Austria, the costs of swallow MRI are 1.7 times higher than of 3D-CT.

Imaging in CT takes only seconds; however, positioning of the patient, oral administration of contrast medium, and intake of the effervescent granules result in a total examination time of about 5 min. By comparison, a study using the same plastic tube technique in swallow MRI reported an average examination time of 32 min [9].

## Limitations

The patient collective in the present study was inhomogeneous due to the different kinds of weight regain procedures the patients had after RYGB. Nevertheless, the pouch volumes were quite uniform, making a comparison possible, as pouches had been resized during the weight regain procedures if necessary.

The high rate of intrathoracic pouch migration detected by 3D-CT and MRT may, in part, be due to the fact that patients were scanned in the supine left anterior oblique position, which results in less downward traction of the pouch than an upright position.

Although this is the first study to compare swallow MRI scans to 3D-CT scans of the pouch in RYGB patients, the number of patients included in this study is too small to draw any definitive conclusions.

## Conclusion

Swallow MRI is a valid method for the evaluation of pouch volume during the different phases of the swallowing process due to its additional dynamic acquisition. There was no inferiority in terms of diagnosis of ITM compared to 3D-CT. Further studies with larger series that would perform swallow MRI in different bariatric procedures are needed to confirm these results.

## References

[1] Angrisani L, Santonicola A, Iovino P, Vitiello A, Higa K, Himpens J, Buchwald H, Scopinaro N (2018). IFSO worldwide survey 2016: primary, endoluminal, and revisional procedures. Obes Surg.

[2] Garvey WT, Garber AJ, Mechanick JI, Bray GA, Dagogo-Jack S, Einhorn D, Grunberger G, Handelsman Y, Hennekens C, Hurley D, McGill J, Palumbo P, Umpierrez G, on behalf of the AACE Obesity Scien (2014). American association of clinical endocrinologists and american college of endocrinology position statement on the 2014 advanced framework for a new diagnosis of obesity as a chronic disease. Endocr Pract.

[3] Felsenreich DM, Langer FB, Bichler C, Kristo I, Jedamzik J, Eilenberg M, Arnoldner MA, Prager G (2019). Surgical therapy of weight regain after Roux-en-Y gastric bypass. Surg Obes Relat Dis.

[4] Yimcharoen P, Heneghan HM, Singh M, Brethauer S, Schauer P, Rogula T, Kroh M, Chand B (2011). Endoscopic findings and outcomes of revisional procedures for patients with weight recidivism after gastric bypass. Surg Endosc.

[5] Abu Dayyeh BK, Jirapinyo P, Thompson CC (2017). Plasma ghrelin levels and weight regain after Roux-en-Y gastric bypass surgery. Obes Surg.

[6] Patel P, Bhogal R, Rajput A, Elshaw A, Sada P, Khan A, Mirza S (2017). Post roux-en-Y gastric bypass complications: a comparative study assessing the clinical effectiveness of oesophagogastroduodenoscopy and oral-contrast swallow. Surgeon.

[7] Baumann T, Grueneberger J, Pache G, Kuesters S, Marjanovic G, Kulemann B, Holzner P, Karcz-Socha I, Suesslin D, Hopt UT, Langer M, Karcz WK (2011). Three-dimensional stomach analysis with computed tomography after laparoscopic sleeve gastrectomy: sleeve dilation and thoracic migration. Surg Endosc.

[8] Robert M, Pechoux A, Marion D, Laville M, Gouillat C, Disse E (2015). Relevance of roux-en-Y gastric bypass volumetry using 3-dimensional gastric computed tomography with gas to predict weight loss at 1 year. Surg Obes Relat Dis.

[9] Arnoldner MA, Kristo I, Paireder M, Cosentini EP, Schima W, Weber M, Schoppmann SF, Kulinna-Cosentini C (2019). Swallowing MRI-a reliable method for the evaluation of the postoperative gastroesophageal situs after Nissen fundoplication. Eur Radiol.

[10] Kulinna-Cosentini C, Schima W, Lenglinger J, Riegler M, Kolblinger C, Ba-Ssalamah A (2012). Is there a role for dynamic swallowing MRI in the assessment of gastroesophageal reflux disease and oesophageal motility disorders?. Eur Radiol.

[11] Forsell P, Hellers G, Laveskog U, Westman L (1996). Validation of pouch size measurement following the Swedish adjustable gastric banding using endoscopy, MRI and barium swallow. Obes Surg.

[12] Baumann T, Kuesters S, Grueneberger J, Marjanovic G, Zimmermann L, Schaefer AO, Hopt UT, Langer M, Karcz WK (2011). Time-resolved MRI after ingestion of liquids reveals motility changes after laparoscopic sleeve gastrectomy--preliminary results. Obes Surg.

[13] Kulinna-Cosentini C, Schima W, Ba-Ssalamah A, Cosentini EP (2014). MRI patterns of Nissen fundoplication: normal appearance and mechanisms of failure. Eur Radiol.

[14] Traser L, Spahn C, Richter B, Baumann T, Schumacher M, Echternach M (2014). Real-time and three-dimensional MRI for diagnosis of pharyngoceles. J Magn Reson Imaging.

[15] Seif Amir Hosseini A, Uhlig J, Streit U, Uhlig A, Sprenger T, Wedi E, Ellenrieder V, Ghadimi M, Uecker M, Voit D, Frahm J, Lotz J, Biggemann L (2019). Hiatal hernias in patients with GERD-like symptoms: evaluation of dynamic real-time MRI vs endoscopy. Eur Radiol.

